# Infected Facial Tissue Fillers Caused by Dental Infection

**DOI:** 10.1155/2021/8661995

**Published:** 2021-11-22

**Authors:** Antoine Berberi, Bouchra Hjeij, Georges Aad, Georges Aoun

**Affiliations:** ^1^Department of Oral and Maxillofacial Surgery, Faculty of Dental Medicine, Lebanese University, Lebanon; ^2^Faculty of Dental Medicine, Lebanese University, Lebanon; ^3^Department of Oral Medicine and Maxillofacial Radiology, Faculty of Dental Medicine, Lebanese University, Lebanon

## Abstract

Injectable dermal fillers are widely used for facial rejuvenation; they help reshape the facial contours by treating volume loss due to aging changes. Facial fillers may become infected following a dental infection. In this report, we present a case of a 44-year-old female patient who presented with a swelling in her upper right buccal region following dental treatment of her second maxillary right premolar. After a thorough history, clinical, and radiological examinations, the diagnosis of infected dermal filler was made. The lesion was treated by association of two antibiotics (ciprofloxacin IM and clindamycin tablets 300 mg), and a complete healing was observed two months after the end of the dental treatments.

## 1. Introduction

Injectable dermal fillers (IDFs) are widely used for facial rejuvenation; they help reshape the facial contours by treating the volume loss due to aging changes [1].

Restoring lost volume has been achieved using autologous fat transfer and human collagen, as well as with nonhuman product sources such as hyaluronic acid (HA), bovine or porcine collagen, and synthetic calcium hydroxylapatite or poly-L-lactic acid. The most common IDFs are the HA derivatives [2].

Usually, IDFs are classified based on their tissue survival durability, which can be divided into four types: (a) the short-term type (up to 6 months), (b) the long-term type (up to 24 months), (c) the semipermanent type that can remain from 2 to 5 years, and (d) the permanent type that can survive longer than 5 years [3, 4].

Although IDFs are usually considered safe, the risk of complications is present; these latter can result as early- or late-onset incidents, and they include granulomas, nodules, material migration, and chronic cellulitis [5–7]. For many authors, homogeneous semipermanent or permanent fillers have been associated with many delayed cases of abscesses and granulomas [8–12].

Moreover, cases of infected facial IDFs originated from odontogenic infections with orofacial swelling have been described [1, 7]. These conditions occurring close to the IDFs should be distinguished from traditional facial cellulitis. Consequently, dental practitioners should be careful when proceeding with any dental procedures in patients with a positive history of facial IDFs.

In this report, we present a case of an infected IDF occurring from an edema of dental origin.

## 2. Case Report

**Figure 1:**
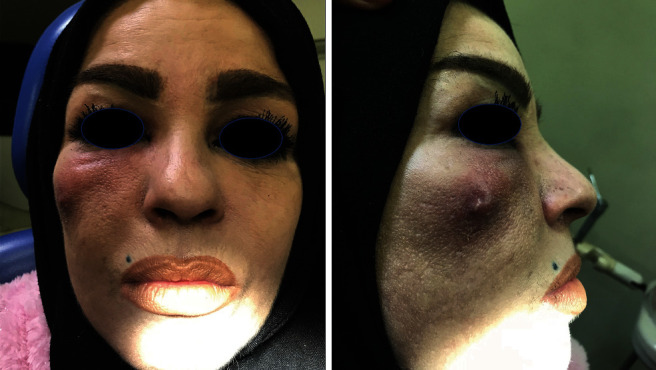
A diffuse, fluctuant, and tender swelling in the right cheek.

A 44-year-old healthy female patient was referred to our department complaining of a swelling in her upper right buccal region following a dental treatment of her second maxillary right premolar (Figure 1).

Clinical examination revealed a diffuse, fluctuant, and tender swelling in her right cheek.

**Figure 2:**
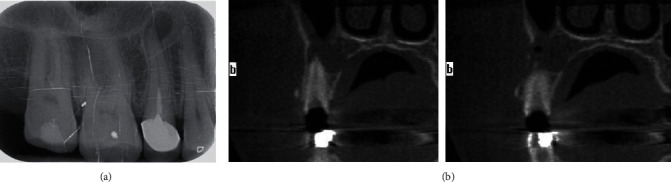
(a) Intraoral radiograph showed radiolucency in relation to the apex of the upper right second premolar. (b) CBCT radiograph showed a radiolucent image related to the apical part of #15 with bone resorption, discontinuity of the buccal and maxillary sinus cortical.

Intraoral radiographs showed radiolucency in relation to the apex of the upper right second premolar (#15) treated endodontically (Figure 2(a)). The upper first premolar (#14) was not vital as the confirmed vitality test.

Cone beamed computed tomography (CBCT) radiograph showed a radiolucent image related to the apical part of #15 with bone resorption discontinuity of the buccal and maxillary sinus cortical (Figure 2(b)).

**Figure 3:**
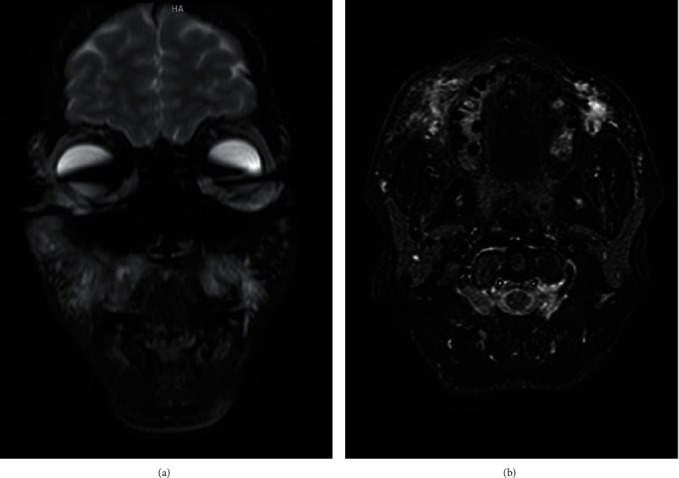
(a) MRI images revealed a subcutaneous high STIR signal fat stranding in both cheeks due to previous filler injection with adjacent mild oedema denoting a reactive inflammatory one. (b) Axial cuts displayed high signal intensity and an expansible lesion in the left cheek, with a buccal bone discontinuity.

MRI images with 1.5 T using routine T1- and T2-weighted spin-echo sequences revealed a subcutaneous high STIR signal fat stranding in both cheeks due to previous filler injection with adjacent mild oedema denoting a reactive inflammatory one. No signs of osteomyelitis were noticed (Figure 3(a)).

Axial cuts displayed high signal intensity and an expansible lesion in the left cheek, with a buccal bone discontinuity. The same high signal was observed in the right cheek (Figure 3(b)).

**Figure 4:**
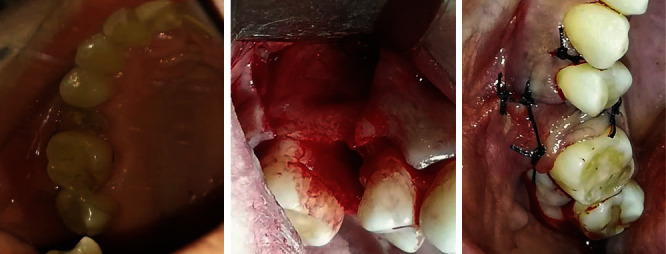
Surgical extraction of tooth 15 with closure of the site to avoid oro-antral communication.

The patient declared having received IDFs in her cheeks bilaterally about 6 years before. She was uninformed of the nature of the filler material used. Treatment plan was proposed as surgical extraction of #15 with closure of the site to avoid an oro-antral communication, as well as a root canal treatment of #14 (Figure 4).

Antibiotics (two times per day of ciprofloxacin IM and clindamycin tablets 300 mg) were prescribed for 10 days.

**Figure 5:**
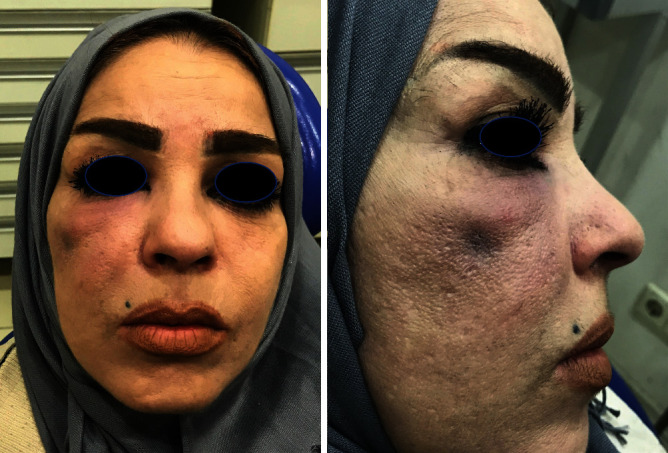
Healing after 15 days.

**Figure 6:**
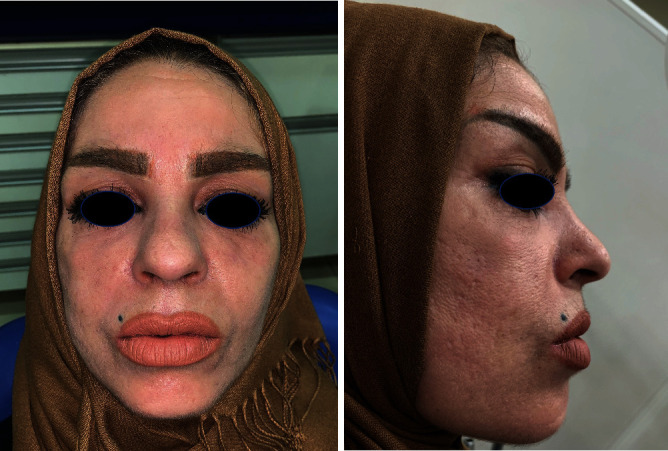
Complete healing two months later.

Healing was observed 15 days after the end of all dental treatments (Figure 5) and completed two months after (Figure 6).

## 3. Discussion

IDFS materials provide an appropriate treatment for the enhancement and rejuvenation of the facial structures. While hydrogels are biocompatible and nontoxic and easily penetrable by nutrients and waste products, making them exceptional growth media for bacteria [13]. Moreover, IDFs are considered safe; nevertheless, the chances of facing some complications still exist as these tissue-injected substances are considered as a foreign body and present an initial challenge to the host side effects [14].

Sclafani and Fagien categorized adverse reactions to fillers into three types: immediate-type (within 24 hours after injection), early-onset type (within 2 weeks), and delayed-type (starting after 2 weeks to years after treatment) complications [8].

Delayed infection events have the same presentation as the early ones; they may present symptoms such as erythema, edema, bruising, itching, pain or tenderness, and nodules or abscesses [4, 15, 16]. Delayed onset of reactions could also be caused by a facial or oral invasive procedure done before the occurrence of the complications [4]. The exact factor causing complications after invasive procedures near filler depots is unknown, but the theory of bacterial contamination of the filler material seems to play an essential role [2]; bacteria are the prime source of biofilms [17]. Biofilms are a structured aggregations of microorganisms encapsulated inside a self-developed polymeric matrix and permanently adherent to a living or inert surface [18]. The free-floating bacteria in tissues become adherent to the foreign body material and consequently develop biofilms. Moreover, bacteria originating from oral conditions and/or procedures can activate the infective response of these biofilms [17]. Once the biofilm has been activated, it leads to acute purulent infection.

Marusza et al., in a case report of delayed infection of injected filler in the cheek, found that remission was observed only after tooth extraction with antibiotic therapy, and they recommend that periodontal health status needed to be assessed prior to facial augmentation to reduce adverse reactions to the filler [19].

Rodriguez et al. noticed that *Mycobacterium chelonae* is one of the bacteria found in a case of cosmetic dermal filler facial infection [20]. For Alijotas-Reig et al. [21] and Christensen et al. [22], *Staphylococcus epidermidis* and *Cutibacterium acnes* (formerly *Propionibacterium acnes*) are responsible of 98% of adverse reactions to dermal fillers. Others found that Staphylococcus aureus and Streptococcus species can be concerned in cases of infection linked to filler injections [23].

Active clinical infections can flare up weeks, months, and even years after initial surgery and can be controlled with antibiotic therapy; however, the underlying biofilm can persist and recur [3, 4, 13].

Many antibiotic groups having the property of adhering to the matrix biofilm such as macrolides, lincosamides, tetracyclines, rifamycins, oxazolidinones, fluoroquinolones, nitroimidazole, and sulphonamides have been recommended [24].

Ferneini et al. suggested the use of oxazolidinone and rifampin as part of combination therapy [16]. Grippaudo et al. recommended clindamycin and levoxacin in a case of filler complications in the perioral region [25].

In our clinical case, the resulting bacteria from the apical infection of the second maxillary premolar moved through the missing buccal bone to the buccal soft tissue and rich injected material in the left cheek. The bacteriological identification of the causative pathogen was not done as the patient did not mention the history of dermal filler.

Our treatment plan was according to Ramzi et al. [7], Ferneini et al. [16], and Marusza et al. [19], and they suggested that dental treatment or tooth extraction improves healing in association with antibiotics.

The missing information from the patient about her dermal filler injection obliged us to perform MRI. The combination of antibiotics and dental treatment allowed us to eliminate the source of infections and to be able to control the spreading of the bacteria in the injected materials.

## 4. Conclusion

Facial IDFs may become infected following a dental infection. The mechanism could be attributed to the bacterial contamination from the mouth through the bloodstream after a dental procedure adjacent to the filler injection that can be activated by the bacterial adherence to the material biofilms. It is essential to raise awareness about the importance of completing dental treatment prior to filler placement, to avoid all risks of complications related to oral conditions. A detailed history of dermal fillers with complete information about the used materials should be known by the patient and declared to the practitioner.

## Data Availability

All data are available in the manuscript.
